# Efficacy of electrical stimulation for post-stroke motor dysfunction: A protocol for systematic review and network meta-analysis

**DOI:** 10.1371/journal.pone.0304174

**Published:** 2024-06-27

**Authors:** Yihao Zhou, Hongyan Zhang, Hong Huo, Siyu Yang, Ying Zhang, Shaojie Cai, Xu Dong, Dongyan Wang

**Affiliations:** 1 Heilongjiang University of Chinese Medicine, Harbin, China; 2 The Second Affiliated Hospital, Heilongjiang University of Chinese Medicine, Harbin, China; University of the Witwatersrand Johannesburg, SOUTH AFRICA

## Abstract

**Objective:**

This study aims to analyze the efficacy and safety of different electrical stimulation treatments for post-stroke motor dysfunction, and to quantitatively analyze the advantages between them and their possible benefits for patients.

**Methods:**

We will systematically search seven databases. All of them will be retrieved from inception to 15, April 2024. Two reviewers will evaluation the risk of bias in all included studies with the version 2 of the Cochrane risk-of-bias assessment tool. Data synthesis will be performed using a random-effects model of network meta-analysis to compare the efficacy and safety of different electrical stimulation therapies. The surface under the cumulative ranking curve was used to indicate the possibility of the pros and cons of the intervention. The strength of evidence will be assessed by the Grading of Recommendations, Assessment, Development, and Evaluation framework.

**Discussion:**

This study will provide evidence that electrical stimulation therapy can effectively improve motor function in stroke patients and will also provide some valuable references for clinical decision-making and treatment guidelines.

**Trial registration:**

PROSPERO registration number: CRD42023459102.

## Introduction

Stroke is the second leading cause of death globally, according to the World Health Organization (WHO) in 2019 [1]. Patients with stroke are susceptible to multiple complications, resulting in survivors facing significant health challenges and loss of a great number of healthy life-years [2, 3]. Moreover, stroke is a cause of long-term disability worldwide, and more than 80% of stroke survivors suffer from hemiplegia, which severely interferes with their daily activities [4, 5]. As we all know, returning to society is the primary rehabilitation purpose of stroke survivors. Therefore, it is very important to find effective and safe rehabilitation methods for patients with post-stroke motor dysfunction.

At present, the rehabilitation methods of hemiplegia after stroke mainly include functional training and physical stimulation. Electrical stimulation is one of the commonly utilized physical rehabilitation methods in stroke patients, with many subtypes and wide applications. Many studies have shown that stimulating paralyzed neuromuscles with different electrical stimulation auxiliary devices can promote the recovery of motor function [6, 7]. The commonly used electrical stimulation methods for rehabilitation of post-stroke motor dysfunction include neuromuscular electrical stimulation [8], functional electrical stimulation [6], stimulation electrode [9], etc. Moreover, acupuncture is recommended by the WHO as a complementary therapy for post-stroke rehabilitation [10]. The researchers combined it with electrical stimulation and derived electroacupuncture therapy, which is now a widely accepted treatment because of its superior efficacy, objectivity, and safety [11]. There is preliminary evidence that electric current could better induce the excitatory response of nerve cells such as neurons, increase the level of brain-derived neurotrophic factor in serum, and accelerate neuronal plasticity and promote neuronal regeneration [6, 12]. Additionally, action potentials generated by external current can cause muscle contraction and then widely activate the response of the sensorimotor nervous system, thus enhancing the potential of motor function reorganization after stroke and play a positive role in the rehabilitation process [13].

Previous pairwise meta-analyses have shown that various electrical stimulation interventions have unique benefits for patients with motor dysfunction after stroke [14–16]. However, the relevant clinical guidelines do not describe in detail how to select and apply them, which mainly depends on the physician’s personal habits and the electrical stimulation devices available in the hospital department. In contrast, network meta-analysis is a novel way to compare direct and indirect evidence and can help researchers gather evidence from multiple randomized controlled trials to determine the probability of the relative efficacy of each intervention, which could help to rank and compare different interventions [17, 18].

To date, two network meta-analyses have separately compared the treatment effects of five electrical stimulation therapies on upper or lower limb dysfunction after stroke [19, 20]. Unfortunately, the electrical stimulation interventions included in these two studies are incomplete, such as electroacupuncture therapy, which is widely used in clinical practice, was not considered. On the other hand, the interventions in these two studies included transcranial direct current stimulation and other limb stimulation therapies, which we believe may be inappropriate because electrical stimulation at different body sites may act in completely different pathways, and this confounding factor may lead to inclusion criteria bias.

Here, we expect to conduct a systematic review and network meta-analysis of the published literature to analyze the efficacy and safety of different electrical stimulation treatments for post-stroke motor dysfunction, and to quantitatively analyze the advantages between them and their possible benefits to patients. The results of this study can provide solid evidence for the selection of treatment strategies in clinical practice and help clinicians and policymakers make better decisions.

## Methods

### Study registration

This protocol for systematic review was carried out in full accordance with the Preferred Reporting Items for Systematic Reviews and Meta-Analyses for Protocols (PRISMA-P) guideline [21] and the PRISMA-extension statement for network meta-analysis [22]. We have registered this protocol on the International Prospective Register of Systematic Reviews (CRD42023459102). The PRISMA-P checklist is shown in S1 File.

### Eligibility criteria

#### Types of studies

Only randomized controlled trials will be included, with no language or region restrictions. Non-randomized controlled trials, case-control studies, cohort studies, case reports, etc. will be excluded.

#### Types of participants

Participants were diagnosed with stroke according to any recognized diagnostic criteria, including ischemic and hemorrhagic, the diagnostic code matched the identifier for stroke in the International Statistical Classification of Diseases. Meanwhile, the participants must also suffer from motor dysfunction on one side. Participants with other causes of limb paralysis will be excluded.

#### Types of interventions

The intervention groups must apply an electrical stimulation therapy to the paralyzed limb, including but not limited to functional electrical stimulation, neuromuscular electrical stimulation, stimulation electrodes, and electroacupuncture. Note that the application of electrical stimulation in areas other than the paralyzed limb, such as transcranial direct current stimulation, will be excluded. However, simultaneous use of transcranial and limb percutaneous electrical stimulation is permitted.

#### Types of control groups

The control groups could gain guideline recommended conventional treatment or exercise rehabilitation therapy. Any type of electrical stimulation therapy applied to the paralyzed limb is not permitted.

#### Types of outcomes

*Primary outcomes*. We selected the Fugl-Meyer Assessment Scale (FMA) [23] as the primary outcome of our network mete-analysis. According to our preliminary search results, FMA is often used to evaluate motor function, balance function, sensory function, etc., after stroke. FMA limb motor function has a total score of 100, including 50 items, each of which is 0–2 points. The higher the FMA score, the better the recovery of motor function, otherwise, it indicates that motor function is seriously impaired.

*Secondary outcomes*. Other data measuring motor function will be used as secondary outcomes likes Modified Ashworth Scale. Additionally, secondary outcomes will include results on activities of daily living and quality of life to provide a more comprehensive assessment of intervention effectiveness, such as modified Barthel Index, quality of life score and safety assessment, etc.

### Data source and search strategy

We will systematically search PubMed, Embase, Cochrane Library, Chinese National Knowledge Infrastructure, VIP Database, Wan-fang Database, Chinese Biomedical Database. All of them will be retrieved from inception to 15, April 2024. The search terms were based on the combination of subject words and free words. Details of the search strategy for the Chinese and English databases are in the S2 File.

### Study selection

**Fig 1:**
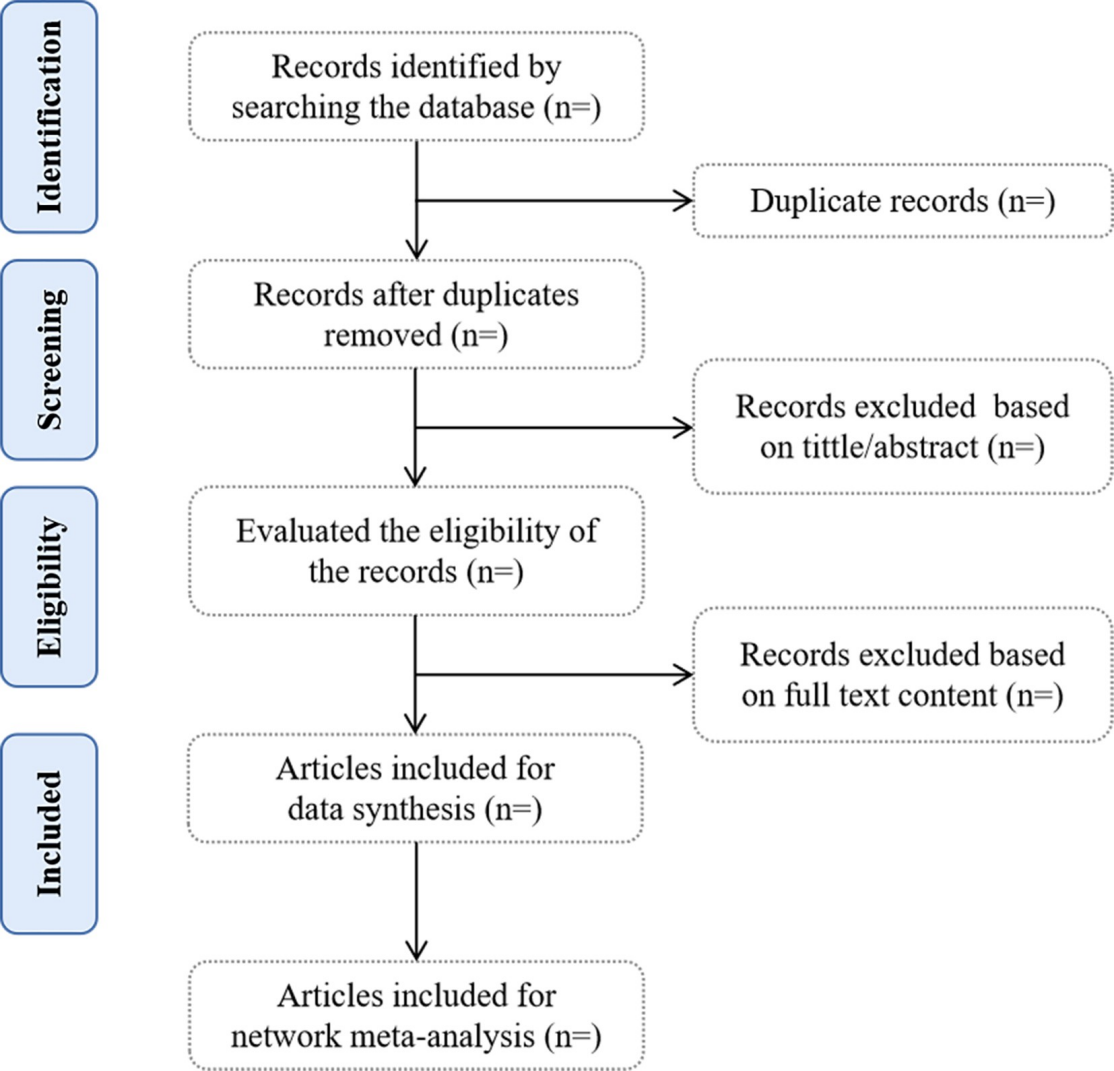
The details of the selection process.

Two reviewers will perform the literature selection independently according to the search strategy. We used EndNote 20 to manage the obtained literature, removing duplicates and then further reading the title, abstract, and full text to screen the literature that met the inclusion criteria. Any disagreement will be adjudicated by a third reviewer. The retrieval flow will be shown in a PRISMA flow chart as Fig 1.

### Data extraction

Two reviewers will independently extract relevant data from all studies ultimately included, following a pre-developed Excel data collection sheet. In case of missing study data, we will contact the authors via email to request complete data. All data will be cross-checked prior to data analysis and any differences will be discussed in the review panel to achieve a consistent result. The extracted data as described below:

1. Study characteristics: author, publication year, country, study design, study centers;
2. Participant characteristics: sample size, gender, age, diagnostic criteria, disease situation (disease course, infarct site, paralyzed limb), use of medications, treatment course, follow-up course;
3. Interventions and comparators details: type of electrical stimulation, duration, frequency, session, and main stimulation parameters;
4. Outcomes: all study measurement data, adverse events, time nodes, etc.

### Risk of bias assessment

Two reviewers will evaluation the risk of bias in all included studies with the version 2 of the Cochrane risk-of-bias assessment tool (RoB 2) [24]. RoB 2 includes the following five projects: bias arising from the randomisation process, bias due to deviations from intended interventions, bias due to missing outcome data, bias in measurement of the outcome, bias in selection of the reported result. The overall predicted direction of bias for outcome as low risk of bias, some concerns and high risk of bias. In order to minimize bias caused by subjective factors of reviewers during the assessment process, detailed guidelines and training will be provided for reviewers before the formal start of the study. Meanwhile, any differences will be discussed and resolved in the review team.

### Data synthesis

The study data were statistically analyzed using Stata 16.0 statistical software (version 17.0; Stata Corporation, College Station, Texas, USA). In the course of the study, the random-effects model was first used to conduct pairwise meta-analysis of the primary outcome, respectively discussing the clinical effects of various electrical stimulation therapy and control group. FMA as an index of continuous variable, we will use standardized mean differences with 95% confidence interval as pooled statistic. *P*-value < 0.05 was considered statistically significant. We will calculate the *I^2^* statistic and *P*-value to assess heterogeneity. Heterogeneity includes methodological heterogeneity and statistical heterogeneity, etc. If *I^2^* > 50%, we will consider the heterogeneity to be present, and we will further discuss the actual sources of heterogeneity, and analyze their potential impact on the findings in the discussion, especially the influence of different study characteristics.

For network meta-analysis, we will choose a random effects mode based on the frequency framework to analyze the direct and indirect evidence of all interventions. The outcome indicators for binary variables will be calculated using odds ratio, while the outcome indicators for continuous variables will be calculated using mean difference or standardized mean difference, both expressed as effect values and 95% confidence interval. First, we will use the "networkplot" command to plot the network relation graph to represent the quantitative relationships between studies and interventions. If the network relation graph can form a closed loop, then the node-splitting method is used to test the inconsistency. Second, data synthesis will be performed using a random-effects model of network meta-analysis to compare the relative efficacy of different electrical stimulation therapies. Finally, the "sucraprob" command was used to rank the efficacy of different interventions, and the cumulative probability graph was drawn. The surface under the cumulative ranking curve (SUCRA) was used to indicate the possibility of the pros and cons of the intervention. Higher SUCRA means that the intervention is more effective. The "netfunnel" command was used to make funnel plots, and publication bias and small sample evaluation were conducted for included studies. We will present the research results in a visual way.

### Sensitivity analysis

We will conduct a sensitivity analysis to verify the robustness and reliability of the results by excluding studies that may have a high risk of bias to determine their potential impact on the results.

### Grading the strength of evidence

We plan to use the Grading of Recommendations, Assessment, Development, and Evaluation (GRADE) framework to assess the strength of evidence for each outcome. The quality grade may be rated down for the following reasons: risk of bias, inconsistency, indirectness, imprecision, publication bias. The strength of evidence has four grades of high, moderate, low, and very low. High quality means that the true effect is close to the estimate of the effect and will be strongly recommended when making clinical decisions. Conversely, the very low quality indicates that the true effect is likely to be substantially different from the effect estimate [25].

### Ethics and dissemination

The data needed for our study came from public databases, and the entire study did not involve patients and the public directly, so the ethical approval was not necessary. The final findings will be peer-reviewed and published in a journal.

## Discussion

The motor dysfunction after stroke seriously affects the patients’ daily life and social activities, and brings great burden to the family and society. Electrical stimulation therapy has been proven to be effective in improving motor function in stroke patients, and some pairwise meta-analyses have also shown that electrical stimulation therapy such as functional electrical stimulation and electroacupuncture is more effective than non-electrical stimulation therapy [16, 26]. This study will analyze the relative efficacy and safety of all currently available electrical stimulation therapies for post-stroke motor dysfunction through a network meta-analysis. This study will provide evidence that electrical stimulation therapy can effectively improve motor function of stroke patients, and provide some valuable references for clinical decision-making and treatment guidelines. According to the study results, doctors can choose more effective electrical stimulation therapy for patients, which will help them recover faster.

## Supporting information

S1 File The PRISMA-P checklist. (DOCX)

S2 File Search strategy for the Chinese and English databases. (DOCX)

## References

[1] Feigin VL, Brainin M, Norrving B, Martins S, Sacco RL, Hacke W, et al. World Stroke Organization (WSO): Global Stroke Fact Sheet 2022. Int J Stroke. 2022;17(1):18–29. doi: 10.1177/17474930211065917 . 34986727

[2] Lam Ching W, Li HJ, Guo J, Yao L, Chau J, Lo S, et al. Acupuncture for post-stroke depression: a systematic review and network meta-analysis. BMC Psychiatry. 2023;23(1):314. doi: 10.1186/s12888-023-04749-1 . 37143014 PMC10161596

[3] Weaver NA, Kuijf HJ, Aben HP, Abrigo J, Bae HJ, Barbay M, et al. Strategic infarct locations for post-stroke cognitive impairment: a pooled analysis of individual patient data from 12 acute ischaemic stroke cohorts. Lancet Neurol. 2021;20(6):448–59. doi: 10.1016/S1474-4422(21)00060-0 . 33901427

[4] Badawi AS, Mogharbel GH, Aljohani SA, Surrati AM. Predictive Factors and Interventional Modalities of Post-stroke Motor Recovery: An Overview. Cureus. 2023;15(3):e35971. doi: 10.7759/cureus.35971 . 37041905 PMC10082951

[5] Lee KB, Lim SH, Kim KH, Kim KJ, Kim YR, Chang WN, et al. Six-month functional recovery of stroke patients: a multi-time-point study. Int J Rehabil Res. 2015;38(2):173–80. doi: 10.1097/MRR.0000000000000108 . 25603539 PMC4415968

[6] Marquez-Chin C, Popovic MR. Functional electrical stimulation therapy for restoration of motor function after spinal cord injury and stroke: a review. Biomed Eng Online. 2020;19(1):34. doi: 10.1186/s12938-020-00773-4 . 32448143 PMC7245767

[7] Marin-Medina DS, Arenas-Vargas PA, Arias-Botero JC, Gomez-Vasquez M, Jaramillo-Lopez MF, Gaspar-Toro JM. New approaches to recovery after stroke. Neurol Sci. 2024;45(1):55–63. doi: 10.1007/s10072-023-07012-3 . 37697027 PMC10761524

[8] Knutson JS, Fu MJ, Sheffler LR, Chae J. Neuromuscular Electrical Stimulation for Motor Restoration in Hemiplegia. Phys Med Rehabil Clin N Am. 2015;26(4):729–45. doi: 10.1016/j.pmr.2015.06.002 . 26522909 PMC4630679

[9] Elsner B, Kugler J, Pohl M, Mehrholz J. Transcranial direct current stimulation (tDCS) for improving activities of daily living, and physical and cognitive functioning, in people after stroke. Cochrane Database Syst Rev. 2020;11(11):CD009645. doi: 10.1002/14651858.CD009645.pub4 . 33175411 PMC8095012

[10] Chavez LM, Huang SS, MacDonald I, Lin JG, Lee YC, Chen YH. Mechanisms of Acupuncture Therapy in Ischemic Stroke Rehabilitation: A Literature Review of Basic Studies. Int J Mol Sci. 2017;18(11):2270. doi: 10.3390/ijms18112270 . 29143805 PMC5713240

[11] Li SS, Hua XY, Zheng MX, Wu JJ, Ma ZZ, Xing XX, et al. Electroacupuncture treatment improves motor function and neurological outcomes after cerebral ischemia/reperfusion injury. Neural Regen Res. 2022;17(7):1545–55. doi: 10.4103/1673-5374.330617 . 34916440 PMC8771092

[12] Kimura T, Kaneko F, Iwamoto E, Saitoh S, Yamada T. Neuromuscular electrical stimulation increases serum brain-derived neurotrophic factor in humans. Exp Brain Res. 2019;237(1):47–56. doi: 10.1007/s00221-018-5396-y . 30306243

[13] Enoka RM, Amiridis IG, Duchateau J. Electrical Stimulation of Muscle: Electrophysiology and Rehabilitation. Physiology (Bethesda). 2020;35(1):40–56. doi: 10.1152/physiol.00015.2019 . 31799910

[14] Sharififar S, Shuster JJ, Bishop MD. Adding electrical stimulation during standard rehabilitation after stroke to improve motor function. A systematic review and meta-analysis. Ann Phys Rehabil Med. 2018;61(5):339–44. doi: 10.1016/j.rehab.2018.06.005 . 29958963

[15] Kristensen MGH, Busk H, Wienecke T. Neuromuscular Electrical Stimulation Improves Activities of Daily Living Post Stroke: A Systematic Review and Meta-analysis. Arch Rehabil Res Clin Transl. 2022;4(1):100167. doi: 10.1016/j.arrct.2021.100167 . 35282150 PMC8904887

[16] Beijora AC, Back AP, Frez AR, Azevedo MRB, Bertolini GRF. Peripheral electrical stimulation on neuroplasticity and motor function in stroke patients: a systematic review and meta-analysis. Neurol Res. 2023;45(12):1111–26. doi: 10.1080/01616412.2023.2257419 . 37732768

[17] Rouse B, Chaimani A, Li T. Network meta-analysis: an introduction for clinicians. Intern Emerg Med. 2017;12(1):103–11. doi: 10.1007/s11739-016-1583-7 . 27913917 PMC5247317

[18] Yang A, Pechlivanoglou P, Aoyama K. Interpreting and assessing confidence in network meta-analysis results: an introduction for clinicians. J Anesth. 2022;36(4):524–31. doi: 10.1007/s00540-022-03072-5 . 35641661 PMC9338903

[19] Tang Y, Wang L, He J, Xu Y, Huang S, Fang Y. Optimal Method of Electrical Stimulation for the Treatment of Upper Limb Dysfunction After Stroke: A Systematic Review and Bayesian Network Meta-Analysis of Randomized Controlled Trials. Neuropsychiatr Dis Treat. 2021;17:2937–54. doi: 10.2147/NDT.S332967 . 34552328 PMC8450164

[20] Fang Y, Li J, Liu S, Wang Y, Li J, Yang D, et al. Optimization of electrical stimulation for the treatment of lower limb dysfunction after stroke: A systematic review and Bayesian network meta-analysis of randomized controlled trials. PLoS One. 2023;18(5):e0285523. doi: 10.1371/journal.pone.0285523 . 37167257 PMC10174537

[21] Shamseer L, Moher D, Clarke M, Ghersi D, Liberati A, Petticrew M, et al. Preferred reporting items for systematic review and meta-analysis protocols (PRISMA-P) 2015: elaboration and explanation. BMJ. 2015;350:g7647. doi: 10.1136/bmj.g7647 . 25555855

[22] Hutton B, Salanti G, Caldwell DM, Chaimani A, Schmid CH, Cameron C, et al. The PRISMA extension statement for reporting of systematic reviews incorporating network meta-analyses of health care interventions: checklist and explanations. Ann Intern Med. 2015;162(11):777–84. doi: 10.7326/M14-2385 . 26030634

[23] Sullivan KJ, Tilson JK, Cen SY, Rose DK, Hershberg J, Correa A, et al. Fugl-Meyer assessment of sensorimotor function after stroke: standardized training procedure for clinical practice and clinical trials. Stroke. 2011;42(2):427–32. doi: 10.1161/STROKEAHA.110.592766 . 21164120

[24] Sterne JAC, Savovic J, Page MJ, Elbers RG, Blencowe NS, Boutron I, et al. RoB 2: a revised tool for assessing risk of bias in randomised trials. BMJ. 2019;366:l4898. doi: 10.1136/bmj.l4898 . 31462531

[25] Puhan MA, Schunemann HJ, Murad MH, Li T, Brignardello-Petersen R, Singh JA, et al. A GRADE Working Group approach for rating the quality of treatment effect estimates from network meta-analysis. BMJ. 2014;349:g5630. doi: 10.1136/bmj.g5630 . 25252733

[26] Zhang J, Zhu L, Tang Q. Electroacupuncture with rehabilitation training for limb spasticity reduction in post-stroke patients: A systematic review and meta-analysis. Top Stroke Rehabil. 2021;28(5):340–61. doi: 10.1080/10749357.2020.1812938 . 32845210

