# Supplementary material for: Efficacy of electrical stimulation for post-stroke motor dysfunction: A protocol for systematic review and network meta-analysis
Source: PLoS One. 2024 Jun 27;19(6):e0304174. doi: 10.1371/journal.pone.0304174 (PMC11210817; doi:10.1371/journal.pone.0304174)
Supplement: S2 File — (DOCX) [file pone.0304174.s002.docx]

**Table 1. Search strategy for the English databases.**

| **Order** | **strategy** |
| --- | --- |
| #1 | (Stroke[MeSH]) OR (Cerebrovascular Accident[Title/Abstract]) OR (CVA[Title/Abstract]) OR (cerebral infarction[Title/Abstract]) OR (Brain Vascular Accident[Title/Abstract]) OR (Apoplexy[Title/Abstract]) |
| #2 | (Hemiplegia[MeSH Terms]) OR (Paralysis[Title/Abstract]) OR (Motor function[Title/Abstract]) OR (Dysfunction[Title/Abstract]) OR (palsy[Title/Abstract]) |
| #3 | (Electric Stimulation[MeSH Terms]) OR (Electrical Stimulation[Title/Abstract]) OR (Electrotherapy[Title/Abstract]) OR (neuromuscular electrical stimulation[Title/Abstract]) OR (NMES[Title/Abstract]) OR (functional electrical stimulation[Title/Abstract]) (FES[Title/Abstract]) OR (Stimulation electrode[Title/Abstract]) OR (Transcutaneous electrical stimulation[Title/Abstract]) OR (Transcutaneous electric stimulation[Title/Abstract]) OR (Transcutaneous electrical nerve stimulation[Title/Abstract]) OR (TENS[Title/Abstract]) OR (Percutaneous electric nerve stimulation[Title/Abstract]) OR (Transcutaneous electrical acupoint stimulation[Title/Abstract]) OR (TEAS[Title/Abstract]) OR (Electrical muscle stimulation[Title/Abstract]) OR (Electric muscle stimulation[Title/Abstract]) OR (Transdermal electrostimulation[Title/Abstract]) OR (Electroacupuncture[Title/Abstract]) OR (Electrical acupuncture[Title/Abstract]) OR (Electric acupuncture[Title/Abstract]) OR (EA[Title/Abstract]) |
| #4 | (Randomized controlled trial[Publication Type]) OR (Semi-randomized controlled trials[Publication Type]) OR (RCT randomized controlled[Publication Type]) OR (Random allocation[Title/Abstract]) OR (Clinical trial[Title/Abstract]) OR (Randomi*[Title/Abstract]) |
| #5 | #1 AND #2 AND #3 AND #4 |

**Table 2. Search strategy for the Chinese databases.**

| **Order** | **strategy** |
| --- | --- |
| #1 | 中风 + 脑卒中 + 卒中 + 脑梗死 + 脑梗塞 + 脑缺血 + 脑出血 + 脑血管病 + 缺血性卒中 + 出血性卒中 |
| #2 | 瘫 + 偏瘫 + 瘫痪 + 活动不利 + 运动障碍 + 肢体功能 + 肢体障碍 + 运动功能障碍 |
| #3 | 电刺激 + 电疗 + 神经电刺激 + 肌肉电刺激 + 神经肌肉电刺激 + 功能性电刺激 + 刺激电极 + 经皮电刺激 + 经皮穴位电刺激 + 经皮神经电刺激 + 电针 + 中频电疗仪 |
| #4 | #1 AND #2 AND #3 |
